# Schisandrin B Alleviates Diabetic Cardiac Autonomic neuropathy Induced by P2X7 Receptor in Superior Cervical Ganglion via NLRP3

**DOI:** 10.1155/2023/9956950

**Published:** 2023-01-10

**Authors:** Zhihua Zhang, Hongmin Guo, Zihui Hu, Congfa Zhou, Qixing Hu, Hao Peng, Gan Tang, Zehao Xiao, Lingzhi Pi, Guilin Li

**Affiliations:** ^1^Queen Mary School, Medical School of Nanchang University, 461 Bayi Road, Nanchang, Jiangxi 330006, China; ^2^Department of Physiology, Medical School of Nanchang University, 461 Bayi Road, Nanchang, Jiangxi 330006, China; ^3^Department of Anatomy, Medical School of Nanchang University, 461 Bayi Road, Nanchang, Jiangxi 330006, China; ^4^School of Basic Medicine, Medical School of Nanchang University, 461 Bayi Road, Nanchang, Jiangxi 330006, China

## Abstract

Diabetic cardiovascular autonomic neuropathy (DCAN) is a common complication of diabetes mellitus which brings about high mortality, high morbidity, and large economic burden to the society. Compensatory tachycardia after myocardial ischemia caused by DCAN can increase myocardial injury and result in more damage to the cardiac function. The inflammation induced by hyperglycemia can increase P2X7 receptor expression in the superior cervical ganglion (SCG), resulting in nerve damage. It is proved that inhibiting the expression of P2X7 receptor at the superior cervical ganglion can ameliorate the nociceptive signaling dysregulation induced by DCAN. However, the effective drug used for decreasing P2X7 receptor expression has not been found. Schisandrin B is a traditional Chinese medicine, which has anti-inflammatory and antioxidant effects. Whether Schisandrin B can decrease the expression of P2X7 receptor in diabetic rats to protect the cardiovascular system was investigated in this study. After diabetic model rats were made, Schisandrin B and shRNA of P2X7 receptor were given to different groups to verify the impact of Schisandrin B on the expression of P2X7 receptor. Pathological blood pressure, heart rate, heart rate variability, and sympathetic nerve discharge were ameliorated after administration of Schisandrin B. Moreover, the upregulated protein level of P2X7 receptor, NLRP3 inflammasomes, and interleukin-1*β* in diabetic rats were decreased after treatment, which indicates that Schisandrin B can alleviate the chronic inflammation caused by diabetes and decrease the expression levels of P2X7 via NLRP3. These findings suggest that Schisandrin B can be a potential therapeutical agent for DCAN.

## 1. Introduction

Diabetic cardiovascular autonomic neuropathy (DCAN) is a serious and common complication of diabetes mellitus (DM), which may affect the prognosis of disorders of autonomic nerve fibers and cardiovascular damage [1]. Previous research has reported that the clinical manifestations of cardiovascular autonomic neuropathy include myocardial ischemia, exercise intolerance, resting tachycardia, and orthostatic hypotension [2, 3]. The prevalence rate of DCAN is 2%-91% in type 1 DM and 25%-75% in patients with type 2 DM. Once diagnosed as DCAN, whether the type 1 or type 2 DM, the five-year mortality rate is 16%-50%, most of which can be attributed to sudden cardiac death [4]. However, treating diabetic complications including DCAN brings a tremendous economic burden to society [5], and the 5-year mortality rate of DCAN is five times higher for patients with DCAN than for patients without it. To date, there was no efficient and systemic drug in the treatment of DCAN.

The pathogenesis of DCAN is related to the dysregulation of superior cervical ganglion (SCG), which is the biggest sympathetic trunk ganglion and an important element of the autonomic nervous system [6, 7]. There is a mutual connection between the cardiac sensory afferent neurons and the superior cervical sympathetic ganglion neurons [8]. The nociceptive signals of myocardial ischemia are transmitted to the SCG via afferent fibers of sympathetic nerve, and the efferent signals from central nervous system are conducted through the postganglionic fibers to modulate the function of the cardiovascular system. Nociceptive signals of acute myocardial ischemia and hypoxia in patients with DCAN can enhance the excitability of the heart through the postganglionic sympathetic nerve, resulting in an increase of heart rate, blood pressure, and cardiac sympathetic nerve excitability, which may cause severe injury to patients with myocardial ischemia [4, 9]. In addition, the dysfunction of SCG in patients with DCAN may be related to high expressions of some receptors which participate in the regulation of cardiovascular system and modulating the expression of these receptors maybe a possible way for the treatment of DCAN [10].

Both type 2 DM and nerve injury have been found to be related to chronic inflammatory responses, which can initiate the expression of large number of P2 receptors [11]. P2X receptor is a group of trimeric ligand-gated ion channel gated by ATP [12]. Activated P2X receptor enables flow of receptors present on many cell types. It facilitates the flow of Na+, Ca2+, and K+ across the cell membrane and mediates a series of biological functions [13, 14]. P2X7 receptor is a kind of P2X receptor, which is widely distributed in many organs including SCG. Because the SCG is a key part of autonomic nerve and cardiovascular system, the postganglionic sympathetic nerve endings of the superior cervical sympathetic nerve distribute in the heart and coronary vessels, and the overexpression of P2X7 receptor in the SCG can disturb the function of cardiovascular system [15]. Previous studies found that P2X7 receptor overexpressed in the SCG of rats with DCAN, also suggesting that the P2X7 receptor in the SCG mediates the pathological changes of cardiac sympathetic postganglionic excitatory reflex mediated by myocardial ischemia [16, 17]. The detection of P2X7 receptor expression in SCG may be used as a basis for the diagnosis of cardiovascular sympathetic neuropathy. Recently investigators have examined the effect of brilliant blue G (antagonist of P2X7 receptor) on the SCG and found that these drugs could inhibit the nociceptive signaling induced by myocardial ischemic injury and offer the cardioprotective action. Thus, it was hypothesized that some other drugs might be selected to treat DCAN mediated by P2X7 receptor of SCG.

Schisandra chinensis is a widely used natural compound of traditional Chinese medicine which can inhibit proliferation of tumor tissue as well as prevent memory deficiency [18]. Schisandrin B is a bioactive compound of Schisandra chinensis, and recent studies have shown that Schisandrin B can provide antioxidant, anti-inflammatory, anti-immune, and cardioprotective effects, its biosafety has also been proven [18–21]. As an empirical traditional Chinese medicine, Schisandrin B may have some complex mechanism of action which has not been elucidated, but recent studies have found the related pathway of Schisandrin B. It may reduce the expression of P2X7 protein which contributes to the activation of NLRP3 inflammasome and the production of the mature IL-1*β*, alleviating neuroinflammation [22, 23]. The existing treatment of DCAN is mainly focused on reducing blood glucose before adjuvant treatment for neurological disorders. Previous studies have shown that the pathogenesis of autonomic neuropathy may involve oxidative stress and inflammatory response [9]. As mentioned before, Schisandrin B was reported to mitigate myocardial injury in cardiovascular disease through its anti-inflammatory and antioxidative effect. It also possesses minimal toxicity and is difficult to be metabolized, which makes it worthy for studying its clinical application.

However, there has been little evidence for the exact mechanism and impact of Schisandrin B on cardiovascular system of patients with DCAN. In this study, because of the anti-inflammatory and cardioprotective effects of Schisandrin B, we aimed to investigate if Schisandrin B could alleviate the symptom induced by DCAN. In addition, because of the role of P2X7 receptor in cardiovascular system, P2X7 as a possible marker in the diagnosis of cardiovascular sympathetic neuropathy is further confirmed, it is of great importance to explore the pathway of Schisandrin B as a new treatment agent in affecting P2X7 receptor so as to elucidate the possible effect of Schisandrin B on DCAN.

## 2. Materials and Methods

### 2.1. Experimental Animals and Grouping

Male Sprague-Dawley rats (180 g–220 g) provided by Laboratory Animal Center of Medical College of Nanchang University were used for the studies. The animal used in this experiment was reviewed and approved by Animal Care Committee of Nanchang University.

**Figure 1:**
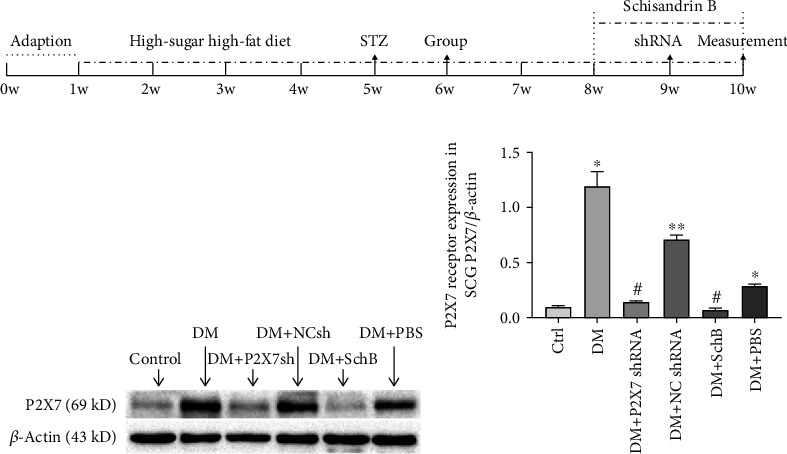
Effect of Schisandrin B on expression levels of P2X7 receptor in SCG of DM rats. (a) The schedule of experiment process. (b) The protein level of P2X7 protein in all group was measured by Western blotting. (c) The relative expression level of P2X7 protein to individual *β*-actin internal control was shown. Data are mean ± SD from four independent experiments. (^∗^*P* < 0.05, ^∗∗^*P* < 0.01 compared with the control group; #*P* < 0.05 compared with the DM group).

Rats in control group (Ctrl, *n* = 8) were given water and normal diet (53% carbohydrate, 23% protein, and 5% fat). High-fat and high-sugar food (77.8% normal diet, 10% sugar, 10% lard oil, 2% cholesterol, and 0.2% sodium cholate) was given to rats for 4 weeks to make type 2 diabetic model rats, and then, rats were given streptozotocin (STZ, 30 mg/kg) by intraperitoneal injection for making diabetic model [24]. Blood glucose was measured to test whether rats have become diabetic after a week of STZ injection. The standard of type 2 diabetic rats is the fasting blood glucose ≥ 7.8 mM or postprandial blood glucose ≥ 11.1 mM [25]. Hyperglycemic rats were divided into 5 groups (*n* = 8 in each group): DM group (type 2 diabetes mellitus), DM + P2X7 shRNA group, DM + NC shRNA group (negative control), DM + Schisandrin B group, DM + PBS group. Rats in DM + shRNA/DM + NC shRNA group were injected P2X7 shRNA/NC shRNA into the SCG. The P2X7 shRNA and NC shRNA were provided by Bio-transduction Lab Company of Wuhan (Wuhan, China), which was mixed with the Entranster™—in vivo transfection reagents (Engreen Company, Beijing, China) [26]. Following the procedure of transfection reagent, each rat in the DM + P2X7 shRNA group was given 5 *μ*g P2X7 shRNA with transfection reagent once only on the first day after grouping. The same dose of scramble shRNA and transfection reagent was given to the DM + NC shRNA group. Rats in the Schisandrin B group were given 3.3 ml/kg Schisandrin B solution by intraperitoneal injection every day in the first two weeks (Figure 1(a)). The same volume of PBS was used in the DM + PBS group. The rats were sacrificed under the anesthesia of intraperitoneal injection of sodium pentobarbital four weeks after grouping for subsequent studies.

### 2.2. Measurement of Blood Pressure and Heart Rate

The softron BP-2010 Blood Pressure Meter was used to measure the heart rate and blood pressure. The heart rate, systolic blood pressure (SBP), mean blood pressure (MBP), and diastolic blood pressure (DBP) of six groups of rats were displayed on the screen by using indirect tail-cuff method according to the instructions.

### 2.3. Measurement of Electrocardiogram and Sympathetic Nerve Activity

Both electrocardiogram (ECG) and sympathetic nerve discharge (SND) were measured by using the RM6240B Data Acquisition and Analysis System of Biological signal (Chengdu Instrument Factory, Chengdu, China). 3% sodium pentobarbital (3 ml/kg) was given by intraperitoneal injection to achieve general anaesthesia. Three electrodes connected to the RM6240B system were fixed under the skin of both upper limbs and right lower limb. For measuring SND, the cervical sympathetic nerve was found and attached to silver electrodes which were connected to the RM6240B system. To reduce the interference, the nerve was isolated from muscle and immersed with paraffin. The settings for measuring SND were as follows: recording sensitivity (25-50 *μ*V), scanning speed (1.0 s/div), power gain (50 *μ*V), and time constant (0.001 s), and frequency filtering (20 kHz) [27].

### 2.4. Western Blotting

After measurement of SND, following the cervical sympathetic nerve to the cranial end, the SCG can be seen behind the bifurcation of the common carotid artery. The SCG can be taken out by cutting the cephalic and centripetal nerves connected to the SCG with ophthalmic scissors, isolated SCGs were washed with PBS smoothly [28, 29]. To extract P2X7 protein, the SCG sample was lysed by mechanical disruption in RIPA buffer on ice. The extract was centrifuged at 12000 × g for 15 min at 4°C. Then, the supernatant was stored at -20°C for the further study. For western blotting with 10 *μ*g protein being used to perform electrophoresis, the sample was loaded on 10% SDS-polyacrylamide gels making by PAGE Gel Quick Preparation Kit (10%; Yeasen Biotech. Co.) according to the instruction, which was more stable and took less time compared to nonfat dry milk. 10 *μ*g protein was used to perform electrophoresis, and then transferred to PVDF membrane which was blocked with protein free rapid blocking buffer (1×; Shanghai EpiZyme Biotech. Co.) at room temperature for 15 min, then incubated with the primary rabbit antibodies against P2X7 (1 : 2000; Abcam International, Inc. USA), IL-1*β* (1 : 500; Affinity, Golden, CO, USA), and NLRP3(1 : 1000; Abcam International, Inc. USA) in western specific antibody dilution buffer (AR1017; Boster Biotech. Co.) at 4 C overnight. Then, it was washed in TBST and incubated in a second antibody solution, goat anti-rabbit IgG (1 : 2000, Beijing Zhongshan Biotech. Co.) in western specific antibody dilution buffer for 2 h at room temperature. The membrane was incubated in electrochemiluminescence (ECL) for 3 min before being put into a chemiluminescence gel imaging system (XRS+, Bio-Rad Company, USA) to detect protein signal. We cleared the membrane by stripping buffer redo blocking and incubation with mouse anti-*β* actin (1 : 2000, A3854; Sigma-Aldrich, St. Louis, MO, USA) to detect protein level of internal control. The expression levels of the P2X7 protein and *β*-actin were quantitated with the Image-Pro software.

### 2.5. Immunohistochemistry

SCGs extracted from rats were washed in phosphate-buffered saline (PBS), and then fixed in 4% paraformaldehyde (PFA) for 24 h at 4°C. The tissues were transferred to 20% sucrose in 4% PFA and kept overnight. Tissues were embedded in OCT and frozen to solid at -80°C, then cut into 8 *μ*m thick with a frozen slicer. Sections were attached on the glass slides and then stored at −20°C. Before the double-label immunofluorescence staining for observation of coexpression of P2X7 and GS, sections were restored to room temperature and washed in PBS and immersed with PFA for 15 min. Following triple washing by PBS, sections were punched with 0.3% Triton X-100 for 10 min at room temperature. After 30-min incubation with goat serum blocking solution, the sections were incubated with mouse anti-GS (1 : 150, ab64613; Proteintech) and rabbit anti-P2X7 (1 : 150, APR-002; alomone labs) overnight at 4°C in cassette, followed by rinsing and incubating sections with TRITC-labeled goat anti-rabbit IgG (1 : 150, E031320-01; EARTHYX) or FITC-labeled goat anti-mouse IgG (1 : 150, E031210-01; EARTHYX) for 1 h at 37°C. The sections were washed and mounted with an antfluorescent quenching agent. The images were captured by fluorescence microscopy (Olympus DP72, Japan) under the same exposure, light, and contrast conditions. The yellow fluorescence density (coexpression level) of each image was analyzed by the Image-Pro Plus software and the region of interest was selected.

### 2.6. Statistical Analysis

All data were presented as mean ± SD (standard deviation). Differences between groups were determined by analysis of variance (ANOVA) followed by LSD post hoc tests using the SPSS 25.0 software (IBM, Chicago, IL, USA). *P* value <0.05 was considered statistically significant.

## 3. Result

### 3.1. Effect of Schisandrin B on Expression of P2X7 Protein in SCG

To determine whether the use of Schisandrin B can influence the expression of P2X7 receptor in the SCG, we assessed the levels of P2X7 protein by western blotting (Figure 1(b)). The results were normalized to their individual *β*-actin internal control. Compared with the control group, the integrated optical density (IOD) of P2X7 protein in the SCG of the DM group, DM + NC shRNA group, and DM + PBS group were significantly higher (*P* < 0.05; Figure 1(c)). However, the difference among the control group, DM+ P2X7 shRNA group and DM+ Schisandrin B group was not statistically significant (*P* > 0.05; Figure 1(c)), and IOD of P2X7 protein in the DM + Schisandrin B group was significantly less than that in the DM group, which indicated that Schisandrin B treatment could lead to reduction of P2X7 expression in SCG.

### 3.2. Changes of Sympathetic Nerve Activity

**Figure 2:**
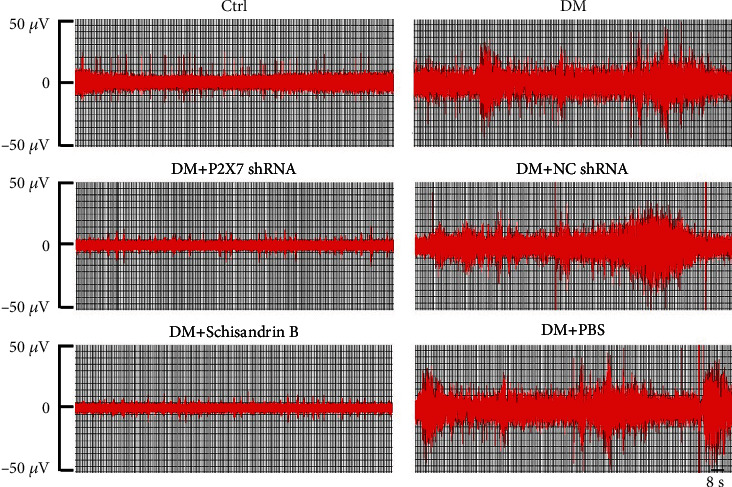
Representative images of the postganglionic cervical sympathetic nerve discharge (SND) of each group. The SND of DM rats was irregular and heightened compared with that in the control group. Sympathetic nerve activity of DM rats was ameliorated after treating with Schisandrin B and shRNA.

Cervical sympathetic nerve discharge of DM rats was significantly strengthened and became more frequent compared to the control group (Figure 2). The SND in DM rats treated with Schisandrin B and shRNA was significantly diminished and became regular compared to that in DM rats treated with NC shRNA and PBS. The results suggested that Schisandrin B could counteract the excitability caused by DCAN, and also improve the sympathetic function.

### 3.3. Changes of Heart Rate and Blood Pressure

The effect of Schisandrin B on the blood pressure and heart rate of diabetic rats are displayed in Table 1. In the DM group, DM + NC shRNA and DM + PBS group, the heart rate (HR), systolic blood pressure (SBP), and mean blood pressure (MBP) were increased in comparison to the control group (*P* < 0.05), indicating compensatory hypertension after myocardial ischemia in the DM rats. The elevated HR, SBP, and MBP declined after using P2X7 shRNA and Schisandrin B (*P* < 0.05), yet the diastolic pressure did not change significantly (*P* > 0.05). The results demonstrated that Schisandrin B could ameliorate the dysregulation of cardiovascular system.

### 3.4. Effect of Schisandrin B on Heart Rate Variability

The effects of Schisandrin B on heart rate variability (HRV) in DM rats are demonstrated in Table 2. The total power frequency (TP), very-low frequency (VLF), low frequency (LF), and high frequency (HF) in the DM group, DM + NC shRNA group and DM + PBS group were reduced significantly in comparison to those in the control group (*P* < 0.01), whereas the LF/HF ratio was increased. Because the LF represents sympathetic activity, HF represents parasympathetic activity, the increase of LF/HF ratio indicates that both sympathetic activity and parasympathetic activity were inhibited and the inhibition of parasympathetic activity was stronger than the sympathetic activity. The decreased TP, VLF, LF, and HF and the increased LF/HF ratio were alleviated in diabetic rats after being treated by P2X7 shRNA and Schisandrin B (*P* < 0.01), indicating that the Schisandrin B improved the pathological sympathetic and parasympathetic activity. In addition, no significant difference was shown among the DM + NC shRNA, DM + PBS group, and DM group, nor among DM + Schisandrin B, DM + P2X7 shRNA group, and control group (*P* > 0.05).

### 3.5. Double-Label Immunofluorescence Staining Detected Expression of P2X7 Receptor and GS in SCG

**Figure 3:**
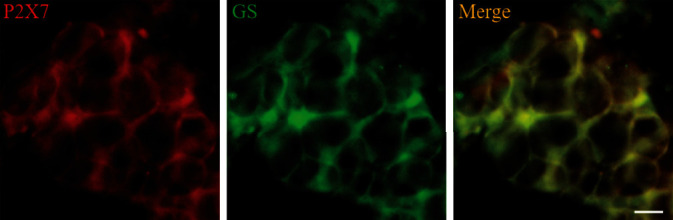
The double-label immunofluorescence staining of P2X7 and GS in SCG. The expression of P2X7 receptor and GS in SCG was detected by double-label immunofluorescence staining. The green signal represents GS staining with FITC, and the red signal indicates P2X7 staining with TRITC. The merged image represents the double staining of P2X7 and GS. Scale bar, 20 *μ*m.

GS as a marker of SGCs was detected with P2X7 receptor by double-label immunofluorescence staining. The GS and P2X7 was surrounded the neurons rather than colocalized with the neuron, which indicated that P2X7 receptor was expressed in the SGCs of SCG (Figure 3).

### 3.6. Effect of Schisandrin B on Expression Level of IL-1*β* in SCG

**Figure 4:**
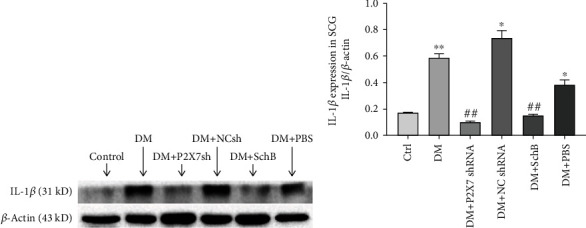
Effect of Schisandrin B on expression levels of IL-1*β* in SCG of DM rats. (a) The protein level of IL-1*β* in all group was measured by western blotting. (b) The relative expression level of IL-1*β* to individual *β*-actin internal control was shown. Values are mean ± SD from four independent experiments. (^∗^*P* < 0.05, ^∗∗^*P* < 0.01 compared with the control group; ##*P* < 0.01 compared with the DM group).

The results from western blot showed that the protein level of inflammatory factors IL-1*β* in the DM group, DM + NC shRNA group and DM + PBS group were upregulated compared with that in the control group (*P* < 0.05). However, administration with P2X7 shRNA and Schisandrin B could reduce the expression of IL-1*β* protein mass in DM group (*P* < 0.01, Figure 4).

### 3.7. Effect of Schisandrin B on Activation of NLRP3 Inflammasome in SCG

**Figure 5:**
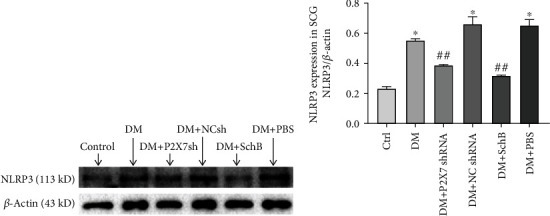
Effect of Schisandrin B on expression levels of NLRP3 in SCG of DM rats. (a) The protein level of NLRP3 in all group was measured by western blotting. (b) The relative expression level of NLRP3 to individual *β*-actin internal control was shown. Values are mean ± SD from four independent experiments. (^∗^*P* < 0.05, ^∗∗^*P* < 0.01 compared with the control group; ##*P* < 0.01 compared with the DM group).

Evidence has suggested that the association between IL-1*β* and P2X7 may be mediated by NLRP3 inflammasome. To determine whether the NLRP3 inflammasome activated in diabetic rats, the expression level of NLRP3 protein were observed by western blot. As shown in Figure 5, DM elicited an apparent upregulation of expression of NLRP3 (*P* < 0.05) in diabetic rats, whereas Schisandrin B and P2X7 shRNA significantly reduced DM induced activation of NLRP3 (*P* < 0.05), indicating that the protective role of Schisandrin B in diabetic model might be partly mediated through inhibiting NLRP3 activation.

## 4. Discussion

DCAN as a cardiovascular disease can be induced by diabetes, and the pathogenesis may be related to P2X receptors. Nociceptive stimulation, nerve injury, and chronic hyperglycemia lead to release of a large number of ATP from nerve endings and stressed nerve cells. Inflammatory cytokines like ATP initiate the expression of P2X receptor, which involves in nociceptive signal transmission and innervation of cardiac autonomic nerve. It is reported that abnormal expression of P2X7 receptor in SCG can induce cardiac autonomic neuropathy by initiating nerve inflammation. Injury of cardiac autonomic nerve and nociceptive signals from ischemic heart caused by DCAN result in increased excitability of heart and abnormal changes of blood pressure, heart rate, and sympathetic activity, causing more severe injury to patients with DCAN [4, 30]. In this experiment, high expression of P2X7 receptor in protein level was observed in rats with DM and the overexpression of P2X7 receptor was downregulated by the administration of P2X7 shRNA and Schisandrin B. The elevated HR, BP, and SND of rats with high P2X7 expression were also mitigated after the treatment of P2X7 shRNA and Schisandrin B. In line with other studies, we found that P2X7 receptor activation induced by DM may participate in the pathogenesis of cardiovascular complications of diabetes mellitus [15, 31].

HRV evaluation is a general method to reflect the status of cardiac autonomic nervous system as well as injury of the sympathetic and parasympathetic nerve. HRV decreases in diabetic rats suggest impairment of the autonomic nervous system and an increased risk for adverse cardiac events [32]. Autonomic fibers that innervate cardiovascular system are damaged by diabetes, and the decreased sympathetic and parasympathetic nerve activity leads to reduced HRV, which is used as an indicator of DCAN. In this study, most of the HRV parameters of diabetic rats decreased as a result of P2X7 receptor activation, illustrating injury of sympathetic and parasympathetic nerve injury. However, LF/HF increased in diabetic rats which suggested that the balance of sympathetic nerve activity and parasympathetic nerve activity was broken to initiate more severe damage to heart [33]. After treating diabetic rats with P2X7 shRNA and Schisandrin B, the abnormal TP, TLF, LF, HF, and LF/HF were alleviated, suggesting that the P2X7 receptor mediates the pathogenesis of diabetic autonomic neuropathy, and that administration of Schisandrin B may relieve DCAN by inhibiting P2X7 receptor expression.

Schisandrin B as a Chinese traditional medicine has not been included in the therapy of DCAN, and most of previous research focused on its antioxidant and antiviral effect [34]. Scientists found that Schisandrin B can reduce inflammation by suppressing the P2X7 receptor expression in acute lung injury [35]. In this study, P2X7 shRNA was used for reducing P2X7 protein expression at SCG as a contrast to the effect of Schisandrin B. Western blotting result showed that increased P2X7 receptor expression in diabetic rats was inhibited by the treatment of P2X7 shRNA and Schisandrin B, which implied that Schisandrin B could reduce P2X7 receptor expression. Therefore, it plays a role in modulation of cardiovascular system. The elevated sympathetic activity, heart rate, blood pressure, and the abnormal traces in ECG induced by overexpression of P2X7 receptor were improved after Schisandrin B administration, suggesting that Schisandrin B can mitigate the damage of autonomic nerves and provide a cardioprotective action.

Chronic inflammation induced by diabetes is related to the excretion of proinflammatory cytokines like IL-1*β*, which exacerbates nerve damage, induces microvascular complications of diabetes, and disturbs the regulation of cardiovascular system [36]. Because overexpression of P2X7 receptor promotes production of these inflammatory factors [37], we speculated that Schisandrin B could alleviate inflammation by reducing P2X7 protein expression [12]. Increased production of IL-1*β* was observed in diabetic rats with high P2X7 expression, and western blot results showed that increased inflammatory cytokines in diabetic rats were mitigated after the treatment of P2X7 shRNA and Schisandrin B, suggesting that P2X7 receptor triggers inflammation in diabetic rats. To further investigate the relationship between P2X7 receptor and inflammation, the P2X7–NLRP3–IL-1*β* pathway was studied, which has been proved to be related to chronic hyperglycemia in preclinical studies [37, 38]. K+ efflux induced by extracellular ATP goes through a purinergic P2X7-dependent channel, resulting in the assembly and activation of the NLRP3 inflammasome [22]. Activation of NLRP3 inflammasome leads to the maturation and release of IL-1*β*, and our data showed that the expression of NLRP3 inflammasome and IL-1*β* increased in diabetic rats. Furthermore, the treatment of P2X7 shRNA and Schisandrin B led to reduction of these cytokines, which is consistent with previous studies that Schisandrin B can reduce inflammation by inhibiting activation of NLRP3 [37].

Compared with previous research involving the effect of long noncoding RNA or scramble siRNA on DCAN, studying on Schisandrin B has more clinical significance because Schisandrin B is given by intraperitoneal injection rather than intrathecal injection [15]. Previous research on Schisandrin B mostly focused on its antioxidant and anticancer effect [39], and our study was the first to apply Schisandrin B for the treatment of cardiovascular diseases. Studies on the molecular pathway of anti-inflammatory function are mostly related to NF-*κ*B and Nrf2, but few are associated with P2X7 receptor [40, 41]. Our study found that the ability of P2X7 receptor to mediate NLRP3 inflammasome involvement identifies a potentially central role for purinergic receptor in the link between Schisandrin B and inflammation in neural tissues. DCAN as a prevalent and dangerous disease had no specific drug at present. Based on the therapeutic effect of Schisandrin B in this experiment, Schisandrin B can be considered as an option for the treatment of DCAN. Finally, Schisandrin B treatment could inhibit the overexpression of P2X7 receptor at SCG in rats with DCAN, alleviate the nerve injury induced by inflammation, offering a cardioprotective action for patients with DCAN. This is the first time that Schisandrin B has been used in the study of DCAN, and future application of Schisandrin B in treatment of DCAN as supplemented regimens needs validation through further study.

## Figures and Tables

**Table 1:** Effect of Schisandrin B on heart rate and blood pressure in rats.

Group	Heart rate (beat/min)	Blood pressure		
		SBP (mmHg)	DBP (mmHg)	MBP (mmHg)
Ctrl	285.18 ± 16.81	121.73 ± 5.27	96.91 ± 10.27	84.82 ± 13.88
DM	384.06 ± 42.24^∗∗^	135.56 ± 6.18^∗∗^	107.19 ± 4.74^∗^	93.06 ± 7.08
DM + P2X7 shRNA	310.35 ± 16.52^##^	117.59 ± 8.25^##^	93.47 ± 4.57^##^	81.47 ± 8.07^#^
DM + NC shRNA	390.55 ± 47.24^∗∗^	134.45 ± 9.56^∗∗^	109.82 ± 18.86^∗∗^	97.45 ± 23.91^∗^
DM + SchisandrinB	321.32 ± 15.27^##^	115.79 ± 10.5^##^	95.53 ± 11.56^##^	85.53 ± 12.66
DM + PBS	347.24 ± 16.37^∗∗^	129.76 ± 10.95^∗^	101.71 ± 14.2	87.76 ± 17.09

Data are mean ± SD from six independent experiments in each group. ^∗^P < 0.05, ^∗∗^P < 0.01 compared with control group; ##*P* < 0.01 compared with DM group.

**Table 2:** Effects of Schisandrin B on heart rate variability in rats.

	TP (ms^2^)	VLF (ms^2^)	LF (ms^2^)	HF (ms^2^)	LF/HF
Ctrl	212.14 ± 18.11	18.45 ± 2.73	57.25 ± 5.24	98.34 ± 10.45	0.57 ± 0.05
DM	35.54 ± 4.11^∗∗^	8.48 ± 0.91^∗∗^	18.23 ± 2.36^∗∗^	9.64 ± 1.33^∗∗^	1.9 ± 0.24^∗∗^
DM + P2X7 shRNA	214.31 ± 19.64^##^	20.03 ± 3.02^##^	58.64 ± 4.86^##^	108.43 ± 11.32^##^	0.55 ± 0.05^##^
DM + NC shRNA	36.24 ± 3.93^∗∗^	9.04 ± 1.04^∗∗^	19.15 ± 3.01^∗∗^	9.27 ± 1.49^∗∗^	2.05 ± 0.31^∗∗^
DM + SchisandrinB	217.02 ± 19.33^##^	21.32 ± 3.26^##^	60.25 ± 5.91^##^	102.31 ± 11.71^##^	0.59 ± 0.08^##^
DM + PBS	36.51 ± 4.31^∗∗^	8.53 ± 0.75^∗∗^	18.04 ± 2.68^∗∗^	8.83 ± 1.62^∗∗^	2.04 ± 0.26^∗∗^

^∗∗^P < 0.01 compared with control group; ##*P* < 0.01 compared with DM group.

## Data Availability

All data generated or analyzed during this study are available from the corresponding author on reasonable request.
